# CircRNA Circ_0000118 Regulates Malignancy of Cervical Cancer Cells by Regulating miR-211-5p/miR-377-3p/AKT2 Axis

**DOI:** 10.1007/s10528-023-10332-w

**Published:** 2023-01-31

**Authors:** Lilan Wu, Huiqin Xiao, Yaqin Hong, Meihua Xie, Yanxia Yu, Lijuan Jiang

**Affiliations:** Department of Obstetrics and Gynecology, Chunan County Traditional Chinese Medicine Hospital, No.1 Xin’an West Road, Chun’an, Hangzhou, 311700 Zhejiang China

**Keywords:** Circ_0000118, Cervical cancer, miR-211-5p, miR-377-3p, AKT2

## Abstract

CircRNAs are implicated in the development of several cancers. Nevertheless, the involvement of circ_0000118 in the development of cervical cancer (CC) remains unclear. Circ_0000118 levels in tumor tissues and cells were examined by qRT-PCR. The function of circ_0000118 in regulating the malignancy of CC cells was investigated using functional assays, including CCK-8, colony formation, transwell, and tube formation experiments. The functional interaction between circ_0000118 and microRNAs were validated by dual-luciferase activity assay and RNA precipitation experiments. In vivo mouse model was employed to assess the effect of circ_0000118 in the tumorigenesis of CC cells. Circ_0000118 was overexpressed in CC cells and tissues. Loss-of-function experiments demonstrated that circ_0000118 knockdown impaired the proliferation and tumor sphere formation, as well as the angiogenic potential of CC cells. RNA interaction experiments confirmed that circ_0000118 sponged miR-211-5p and miR-377-3p. AKT2 was found to be a target gene negatively modulated by miR-211-5p and miR-377-3p. AKT2 overexpression rescued the inhibition of circ_0000118 downregulation on CC cells. Our study suggested that circ_0000118 functions as an oncogenic factor in progression of CC by maintaining AKT2 level through targeting miR-211-5p and miR-377-3p as a ceRNA (competitive endogenous RNA), which provides novel therapeutic target in the management of CC.

**Supplementary Information:**

The online version contains supplementary material available at 10.1007/s10528-023-10332-w.

## Introduction

Cervical cancer (CC) represents a dominant type of malignancy threatening female health (Bray et al. 2018). Human papillomavirus (HPV) infection is one of the major causes triggering CC initiation and progression, which is often transmitted and associated with high-risk sex behavior, immune function suppression, and chlamydia-associated pleural pregnancies (Franco et al. 2001). In spite of the advancement of cancer therapies, CC patients suffer from a low overall survival rate because of cancer metastasis or recurrence (Clark et al. 2016). Exploring the molecular players responsible for CC malignancy and development could lay the foundations for the formulation of novel therapies.

Circular RNAs (circRNAs) are usually produced by back-splicing events during transcription, which are non-coding transcripts characterized by the high stability due to the circular structure (Anastasiadou et al. 2018). CircRNAs are abundantly expressed in eukaryotic cells and show tissue-specific expression pattern (Beermann et al. 2016). Recent studies also unveiled that circRNAs can be packaged in the exosomes as intercellular communication messengers (Pamudurti et al. 2017), and the dysregulation of certain circRNAs has been proposed as the biomarkers for diverse disorders (Patop and Kadener 2018). Besides, cancer-specific serum circRNAs was able to distinguish cancer cases from normal subjects, indicating that liquid biopsy based on cancer-specific circRNAs can be used as a novel diagnostic approach. A recent report demonstrated that SA_circ_0008285 was highly expressed during the progression of CC, revealing a novel regulatory network of SA_circ_0008285 and the downstream microRNAs (Bai and Li 2020).

MicroRNAs (miRNAs) also belong to ncRNAs, which contain 20 to 22 nucleotides (Rupaimoole and Slack 2017). They regulate downstream target expression through modulating the mRNA stability or the translation efficiency (Gebert and MacRae 2019). Recent work suggests that multiple miRNAs were involved in the malignant development of CC, including miR-124, miR-125a, miR-214, and miR-374c (Ruan et al. 2020; Lv et al. 2021; Wang et al. 2020; Huang et al. 2017). However, it is shown that miR-377-3p downregulation is related to the prognosis of CC patients (Zhang et al. 2020). In addition, AKT2 (AKT Serine/Threonine Kinase 2) upregulation plays a critical role in carcinogenesis, metastasis, and angiogenesis, which can be employed as an anti-cancer target in hepatocellular carcinoma (Wang et al. 2016), ovarian cancer (Lin et al. 2018), and CC (Chen et al. 2020). The high expression of AKT2 in CC has also been reported to contribute to the malignant progression (Zhao et al. 2020). However, the mechanism by which AKT2 is overexpressed in CC requires further clarification.

Although it has been reported that circ_0000118 showed high expression in CC (Bai and Li 2020), its functional and regulatory effects need to be elucidated. In this study, we first validated the overexpression of circ_0000118 in CC and its oncogenic role in a mouse xenograft model. High level of circ_0000118 was essential to support the hyperproliferation, invasion, and angiogenesis. We further dissected the downstream targets regulated by circ_0000118 in CC. Our study suggests that circ_0000118 functions as a tumor-promoting molecule in CC by modulating miR-211-5p/miR-377-3p/AKT2 axis.

## Materials and Methods

### Samples and Cell Lines

A total of 78 pairs of tumor samples and the adjacent non-carcinoma control samples were collected by surgery from CC patients in our study. The peripheral blood was also sampled from each individual and the serum was prepared and preserved under − 80 °C till further analysis. The Medical Ethics Committee of Chunan County Traditional Chinese Medicine Hospital approved the experimental protocols involving human subject (approval number 2017-016). The procedures involving human subjects were conducted following the Declaration of Helsinki. The informed consent was signed by all the enrolled subjects.

Normal human cervical epithelial cell line (H8) and HeLa, CaSki, SiHa, and HEK293 cells were acquired from Shanghai Institutes for Biological Sciences of the Chinese Academy of Sciences. All the cell lines were maintained within 90% DMEM (ScienCell) and 10% FBS (ScienCell, USA) under 37 ℃ and 5% CO_2_ conditions. Cells were passed by trypsin digestion every three days or when the density reached 85% confluence. All the experiments were performed using cells before 12 passages.

### Cell Transfection

MiR-211-5p and miR-377-3p inhibitors (GenePharma, Shanghai, China) were used to silence miR-211-5p and miR-377-3p expression, with corresponding inhibitor NC as the controls. Circ_0000118-targeting siRNA sequences (si-circRNA0000118-#1–3: 5′-ACGGGAAAGGTTGAAAGGATT-3′, 5′-GGGAAAGGTTGAAAGGATTGT-3′, 5′-AACGGGAAAGGTTGAAAGGAT-3′, (Ribobio, Guangzhou, China)) were employed to downregulate the circ_0000118. The full length of AKT2 was cloned in the pcDNA3.1 plasmid to overexpress AKT2, with pcDNA3.1 empty vector overexpression control. Cell transfection was conducted by Lipofectamine 3000 (Thermo Fisher Scientific, Shanghai, China) following the recommended protocols of the supplier. 100 nM of siRNA or miRNA mimic/inhibitor was applied 48-h transfection before further experimental studies.

### Subcellular Fractionation

The nuclear and cytoplasmic portions of cells were separated by a Nuclear and Cytoplasmic Extraction Kit (BioGot, Nanjing, China). After first lysis with cytoplasmic buffer, cells were centrifuged at 12,000×*g* for 15 min and the supernatant was harvested as the cytoplasmic part. The resulted pellet was washed twice with the nucleus washing buffer and then collected as the nuclear fraction. Total RNA samples in both nuclear and cytoplasmic portions were collected using Trizol reagent. The relative abundance of each RNA molecule was examined RT-qPCR with U6 as the nuclear marker and GAPDH as the cytoplasmic reference.

### RT-qPCR

Trizol reagent (Yeasen, Shanghai, China) was employed for RNA sample purification from cultured cells or collected peripheral blood following specific protocol. Purified RNA was quality checked with NanoDrop spectrophotometer and RNA samples were utilized for reverse transcription by RevertAid RT Kit (Thermo Fisher Scientific, CA, USA). Gene expression quantification was conducted on the 7900HT Fast qPCR platform (Applied Biosystems, CA, USA) using SYBR green qPCR master mix (YEASEN, Shanghai, China), with the PCR conditions as follows: 95 °C 10 min, 44 rounds of 95 °C 20 s, 60 °C 25 s, and 72 °C 45 s. Gene expression determination was performed by 2^−ΔΔCt^ approach, with beta-actin being the internal reference for mRNA and U6 being the reference for non-coding RNAs. Primers used were circ_0000118, F-GGGCAAAGATGGATTGAAGACA, R-TGCTTCTTCCAAGGCCTTCT; U6: F-TGCGGGTGCTCGCTTCGGCAGC, R-CCAGTGCAGGGTCCGAGGT; Actin: F-CATGTACGTTGCTATCCAGGC, R-CTCCTTAATGTCACGCACGAT; miR-211-5p: F-CAGTTCCCTTTGTCATCCTTC, R-CTCAACTGGTGTCGTGGA; miR-377-3p: F-CAGAGAGGTTGCCCTTG, R-CTCAACTGGTGTCGTGGA; and AKT2: F-AGGCACGGGCTAAAGTGAC, R-CTGTGTGAGCGACTTCATCCT.

### Cell Counting Kit-8 (CCK‑8) Assay

CCK-8 kit (Dojindo, Kumamoto, Japan) was used to determine cell growth ability. After transfection, cells (3000–5000/well) were inoculated into 96-well plates and incubated for indicated durations. At indicated time point, CCK-8 solution (5 μL) was loaded to the cell culture for 2-h incubation. After that the absorbance data were measured at 450 nm using the microplate reader (Biotek, CA, USA).

### Colony Formation Assay

Cells after transfection were plated in 6-well plates (at 2000 cells/well) and fresh medium was replenished every 3 days. After two weeks, fixation was performed by 4% paraformaldehyde for 20 min and cells were labeled with 0.25% crystal violet (Sigma, Germany) for 25 min. Colony formation images were recorded under EVOS M7000 microscope (Thermo Fisher Scientific, CA, USA).

### Tumor Sphere Assay

Tumor spheres were generated by seeding the cells into 12-well plates (at 250 cells/well) coated with 100-μL extracellular matrix (ECM) gel (Corning, CA, USA). Cells were further cultured for 4 days in the incubator until individual spheroids were formed. The efficiency of sphere formation was determined as the ratio of sphere quantity and the seeding number under EVOS M7000 microscope.

### Transwell Invasion Assay

Transwell cassettes (Corning, NY, USA) with matrigel coating were utilized in the invasion experiment. 1 × 10^5^ cells were suspended the culture medium without serum and seeded into the upper cassette. The lower cassette was filled with 400 μL of medium with 15% serum. After 24 h, cells were fixed with cold ethanol for 30 min and stained with 0.25% crystal violet (Sigma, Germany). The cell invading images were captured under EVOS M7000 microscope.

### Dual-Luciferase Reporter Assay

Sequences corresponding to the wild-type (WT) interaction sites or the mutated fragment (MUT) were inserted into the PmirGLO luciferase vector. Cells were co-transfected with the reporter plasmid and miRNA mimic or miR-NC (negative control for miRNA mimic) for 48 h. Cells were then lysed and luciferase activities were determined in the cell lysates using Pierce™ Renilla-Firefly Luciferase Dual Assay Kit (Thermo Fisher Scientific, CA, USA).

### Western-Blotting (WB) Assay

Protein quantification was performed in the cell lysate supernatant by Micro Protein Assay Kit (Thermo Fisher Scientific, CA, USA). 10 μg of total cell protein sample was analyzed through 12% SDS-PAGE. After the transfer to PDVF membrane, the membrane was subjected to 1-h blocking using 5% BSA solution at ambient temperature. Proteins were detected with corresponding antibodies (1:1000 dilutions, all from Abcam) at 4 °C for 18 h. The membrane was further labeled with secondary antibodies conjugated with HRP to detect primary antibodies. Signal development was performed using a chemiluminescent detection kit (Beyotime, Beijing, China) and the images of bands were recorded on the BioRad gel doc system (BioRad, CA, USA).

### Tube Formation Assay

Tube forming experiment was employed to determine the in vitro angiogenesis, with an in vitro angiogenesis assay kit (Abcam, Cambridge, UK). In brief, ECM gel was used to coat 96-well plates for 15 min in the incubator. Cells (1.0 × 10^4^) were added into ECM-coated plates and cultured for 16 h. Tube formation images were recorded under EVOS M7000 microscope. The analysis of tube formation images was performed using ImageJ software (NIH, MD, USA).

### RNA Pull-Down Assay

Streptavidin (Thermo Fisher, USA) was used to treat the biotin-conjugated mutated (MUT) circ_0000118 and wild-type (WT) circ_0000118 for a 12-h period. Afterward, centrifuge at 3000 rpm and then wash with washing solution I for 3 times. At 48-h post-transfection, this work collected cell lysates and cultivated them for a 3-h period using Dynabeads M-280 Streptavidin (Invitrogen) at 4 °C in line with specific instructions. After rinsing thrice using cold solution as well as high-salt buffer (consisting of 500-mM NaCl, 1% Triton X-100, 0.1% SDS, 20-mM Tris–HCl pH 8.0, 2-mM EDTA), the purification of those extracted RNAs was performed with the TRIzol (Invitrogen, Carlsbad, CA, USA) and qRT-PCR assay.

### RNA Immunoprecipitation (RIP) Assay

Cells were lysed using RIP buffer (Yeasen, Shanghai, China) and mixed with 100 μL of protein A/G agarose beads (Yeasen, Shanghai, China), which was pre-labeled with anti-Argonaute 2 antibody or IgG isotype control (FineTest, Wuhan, China). After incubation at 4 °C overnight with rotation, the beads were precipitated by centrifugation and washed thrice with RIP buffer. RNA sample associated with the beads was extracted using Trizol reagent and analyzed by qRT-PCR assay.

### Subcutaneous Xenograft Model

Twelve BALB/c female nude mice (4-week-old) were assigned into sh-NC group_SiHa cells expressing sh-NC (control) and sh-circ_0000118 group_ cells expressing sh-circ_0000118 (sh-RNA targeting circ_0000118), with 6 mice in each group. 2 × 10^6^ cells in PBS were subcutaneously injected in each mouse. Tumor growth was recorded weekly and determined as *V* = 0.5 × length × width^2^. If the tumor size exceeded 2000 mm^3^, the mice were euthanized. At week 5 mice were sacrificed using a CO_2_ chamber at an air rate to fill 40% volume every minute. The terminal death was reassured by no sign of movement and the mice were further cervically dislocated. Xenograft tumor tissues were collected and Ki-67 protein level in the xenograft sections was measured by Immunohistochemistry (IHC). Above animal protocol was approved by the animal welfare Committee of Chunan County Traditional Chinese Medicine Hospital (approval number 2021-D023) and carried out following the institutional guidelines.

### Statistical Analysis

SPSS17.0 software was used to analyze experimental data. Student’s t test was applied for the data analysis between two experimental groups. One-way or two-way ANOVA was adopted for the statistical comparison among 3 or more groups, with Tukey’s test as the post hoc analysis. *P* < 0.05 represents the threshold of significance determination.

## Results

### Circ_0000118 is Highly Expressed in CC Cells and Tumor Sample

**Fig. 1:**
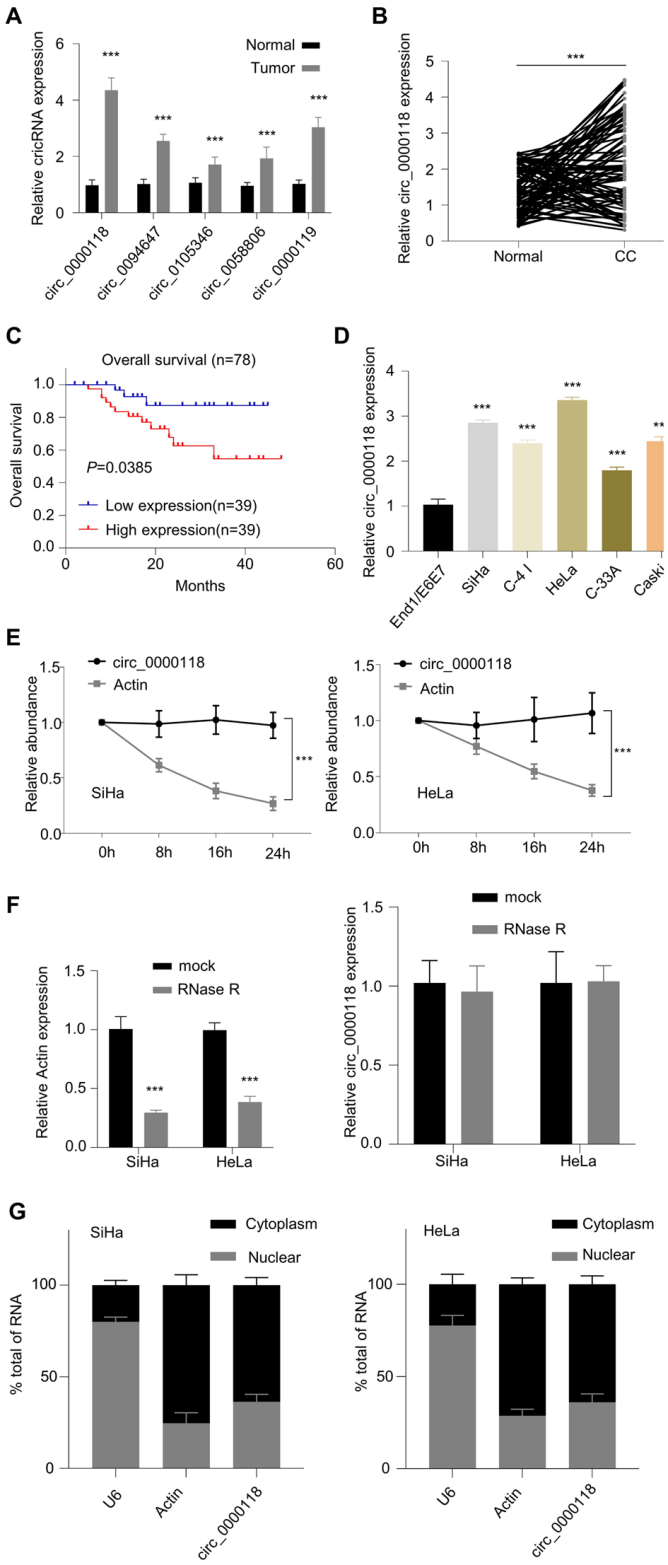
Expression pattern of circ_0000118 levels in cervical cancer cells. **A** Examination of multiple circ_RNAs in CC tumors and normal samples (n = 3 pairs) by qRT-PCR. **B** Circ_0000118 expression in 78 pairs of CC tumor samples and the adjacent normal specimens by qRT-PCR. **C** Kaplan–Meier Curve assessment of the prognosis of CC patients with the high or low circ_0000118 level (n = 39 each). **D** Circ_0000118 expression level in CC cell lines (C-4 I, SiHa, HeLa, Caski, C-33A) as well as normal cervical epithelial cells (End1/E6E7). **E** β-actin and circ_0000118 expression levels at different time points after actinomycin D challenge were measured through qRT-PCR. **F** β-actin and circ_0000118 level detection after RNase R treatment. **G** Relative abundance of circ_0000118 in the cytoplasmic and nuclear portions of CC cells. β-actin serves as the cytoplasmic control and U6 serves as the nuclear control. **P < 0.01, ***P < 0.001

We first assessed the expression of several upregulated circRNAs which were implicated in CC in a previous report (Xu et al. 2020). Among these upregulated circRNAs, we found that circ_0000118 was the mostly upregulated in CC tumors compared to normal samples by qRT-PCR (Fig. 1A). We also collected 78 pairs of tumor samples and the adjacent non-carcinoma control samples from CC patients to profile circ_0000118 expression levels, and there was an upregulation of circ_0000118 expression in in the CC tumor samples compared to the matched non-carcinoma control (Fig. 1B). According to the median circ_0000118 level in CC samples, CC patients were assigned as circ_0000118 low-expression group and high-expression group. It was found that high circ_0087378 level was positively correlated with a larger tumor size, a poorer tumor differentiation, more advanced tumor stages, and the presence of metastasis in cervical cancer patients (Table 1). Furthermore, high level of circ_0000118 was connected to a poorer prognosis in CC patients (Fig. 1C). Additionally, circ_0000118 expression level in CC cell lines (HeLa, Caski, SiHa, C-4 I, C-33A) was significantly higher than that of normal cervical epithelial cells (End1/E6E7) (Fig. 1D). SiHa and HeLa cells with high level of circ_0000118 expression were selected for the subsequent assays.

**Table 1:** Correlative analysis of circ_0000118 level with clinicopathologic parameters of CC patients

Chinicopathological characteristics	Circ_0000118 expression		Total	*χ*^2^	*P*
	Low (n = 39)	High (n = 39)			
Age (year)				0.5098	0.475
≤ 60	24	27	51		
> 60	15	12	27		
Tumor size				8.759	0.003
< 3 cm	28	15	43		
> 3 cm	11	24	35		
Tumor differentiation				5.214	0.0224
Low	12	22	34		
High	27	17	44		
TNM stage				4.336	0.0373
Ι/II	28	19	47		
III/IV	11	20	31		
Lymph node metastasis				5.571	0.0183
Positive	9	19	28		
Negative	30	20	50		

To demonstrate the stability of circ_0000118, we treated SiHa and HeLa cells with actinomycin D to arrest transcription. qRT-PCR analysis showed that β-actin (linear mRNA) level declined following actinomycin D exposure in comparison to the mock group, while circ_0000118 level remained unchanged (Fig. 1E). Similar results were observed upon RNase R digestion of the RNA samples, which demonstrated the resistance of circ_0000118 toward RNase treatment (Fig. 1F). We further performed subcellular fractionation and quantified the relative abundance of circ_0000118 in the subcellular fractions. RT-qPCR results indicated that circ_0000118 was predominantly present in the cytoplasmic fraction in SiHa and HeLa cells (Fig. 1G).

### Knocking Down Circ_0000118 Inhibits the Proliferation, Invasion, and Angiogenesis in CC Cells

**Fig. 2:**
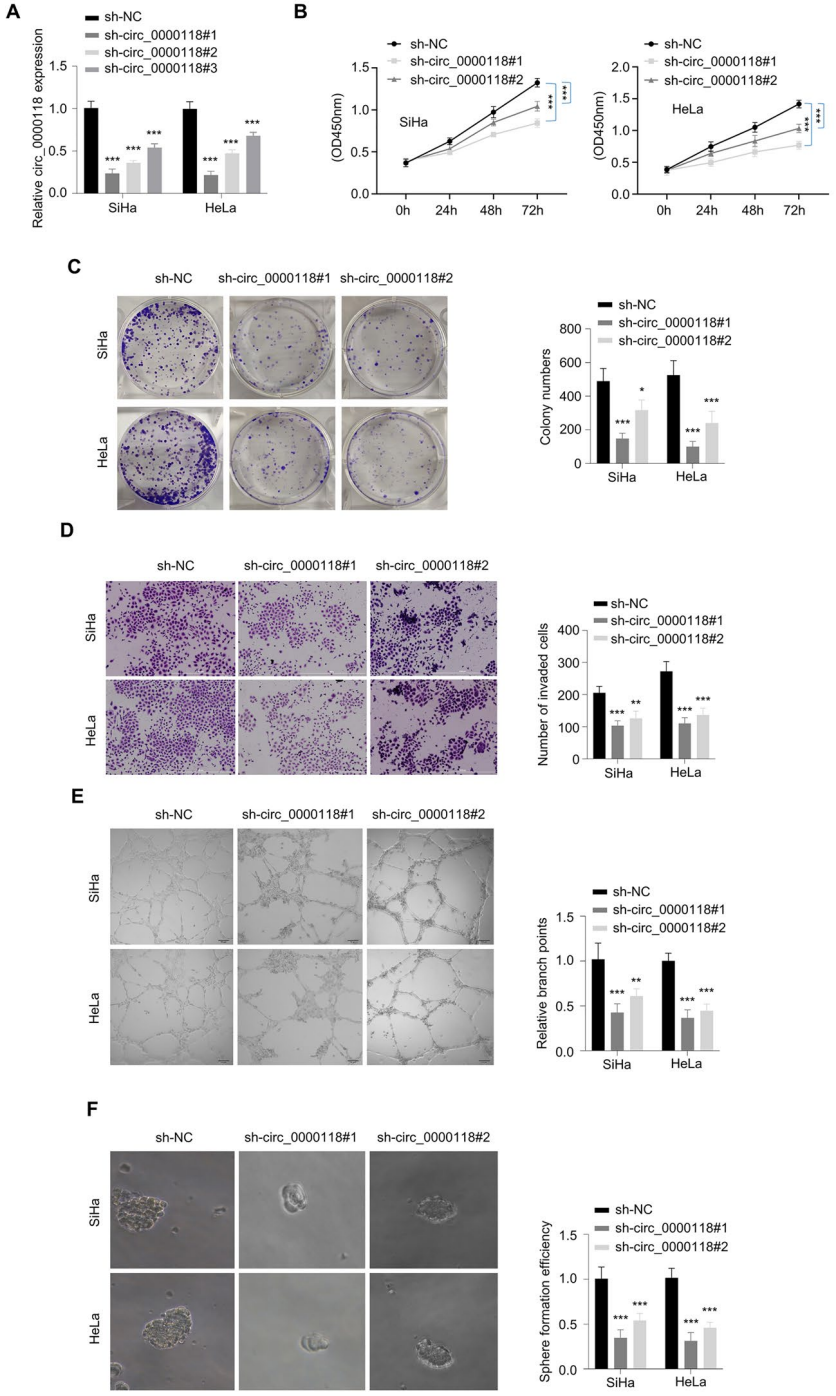
Knockdown circ_0000118 suppresses the cell growth, invasion, and angiogenesis of CC cells. **A** Knockdown efficiency of three sh-RNAs targeting circ_0000118 was validated by qPCR. **B** CCK-8 proliferation experiment was conducted at 0 h, 24 h, 48 h, and 72 h in CC cells upon circ_0000118 downregulation. **C** Colony generation experiment in CC cells upon circ_0000118 downregulation. **D** Invasiveness of CC cells upon circ_0000118 downregulation. **E** Angiogenesis assay in CC cells after circ_0000118 knockdown. **F** 3D sphere tumor assay in CC cells upon circ_0000118 silencing. **P < 0.01, ***P < 0.001

The functional roles of circ_0000118 in CC cells (SiHa and HeLa) were studied by knockdown experiment using sh-RNAs targeting circ_0000118 (sh-circ_0000118 #1, #2, and #3). The transfection of sh-circ_0000118 #1 and #2 showed strong knockdown efficiency and was selected for the knockdown (Fig. 2A). Cell proliferation analysis showed that knocking down circ_0000118 significantly suppressed cell growth (Fig. 2B), which was also supported by the observation that silencing circ_0000118 impaired the colony-forming capacity of CC cells (Fig. 2C). Transwell invasion assay showed that knocking down circ_0000118 undermined the invasiveness in CC cells (Fig. 2D). Tube formation assay further revealed that silencing circ_0000118 impaired the angiogenic potential in CC cells (Fig. 2E). Circ_0000118 knockdown also repressed CC cell proliferation in 3D sphere tumor culture (Fig. 2F). Thus, we concluded that circ_0000118 overexpression is essential to sustain the malignant phenotype in CC cells.

### Circ_0000118 Targeted miR-377-3p and miR-211-5p

**Fig. 3:**
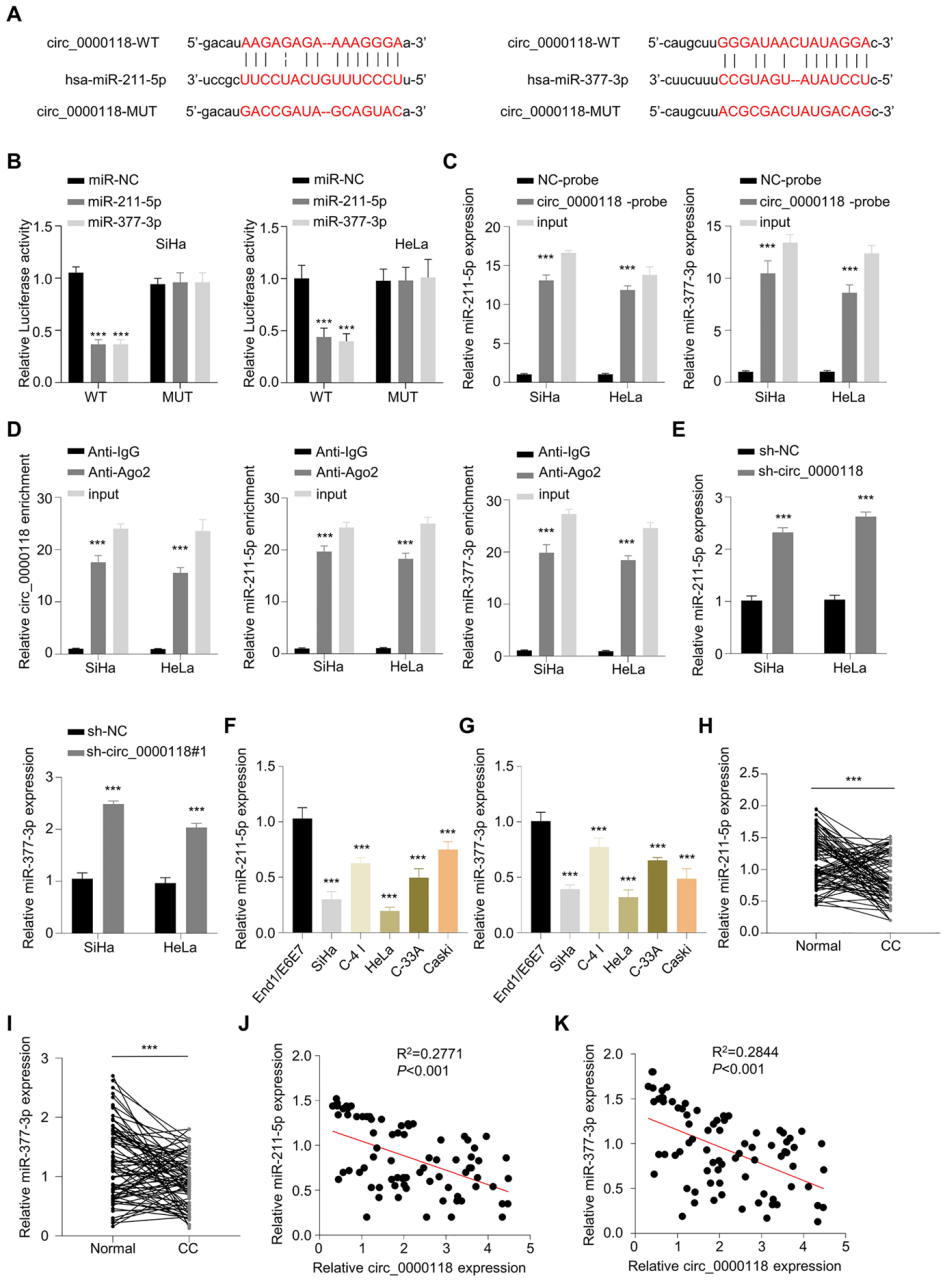
Circ_0000118 targets miR-211-5p and miR-377-3p. **A** Analysis of Starbase online resources revealed the interaction sequences between circ_0000118 and miR-211-5p/miR-377-3p. **B** Dual-luciferase activity assay in CC cells with WT or MUT luciferase vector and miRNA mimic or miR-NC. **C** Biotin-conjugated circ_0000118 precipitated more miRNAs than the negative NC probe. **D** RIP analysis by anti-Argonaute 2 antibody or IgG isotype. **E** miR-211-5p and miR-377-3p levels in CC cells after circ_0000118 knockdown were measured through qRT-PCR. **F**, **G** miR-211-5p and miR-377-3p expression levels in CC cell lines were compared to that of normal epithelial cells (End1/E6E7). **H**, **I** The levels of miR-211-5p and miR-377-3p in 78 CC patient samples and healthy subjects. **J**, **K** Correlative analysis of circ_0000118 level and miR-211-5p/miR-377-3p in 78 CC samples. **P < 0.01, ***P < 0.001

Through the searching in Starbase online resources, there were several miRNA targets for circ_0000118 (Fig. S1A). Through RNA pull-down analysis using biotin-labeled circ_0000118 probe, circ_0000118 could enrich miR-211-5p and miR-377-3p at high level (Fig. S1B). The interacting sites between circ_0000118 miR-211-5p and miR-377-3p were shown (Fig. 3A). Their interactions were verified by luciferase reporter activity assay since miR-211-5p overexpression repressed the WT reporter activity but showed no effect in MUT reporter (Fig. 3B). Further, circ_0000118 probe was able to enrich miR-211-5p and miR-377-3p to a higher level compared with negative control (NC probe) (Fig. 3C). RNA immunoprecipitation (RIP) assay further showed that miR-211-5p, miR-377-3p, and circ_0000118 could be enriched by Ago2 antibody, indicating their interactions in the Ago-containing RNA processing complex (Fig. 3D). Knocking down circ_0000118 caused an increased expression of miR-377-3p and miR-211-5p (Fig. 3E). In addition, miR-377-3p and miR-211-5p levels were downregulated in CC cells (Fig. 3F, G), as well as in CC patient samples when compared to healthy controls (Fig. 3H, I). Importantly, circ_0000118 level displayed a negative correlation with that of miR-211-5p and miR-377-3p in cervical cancer patient samples (Fig. 3J, K). Collectively, these data suggest that circ_0000118 serves as a sponging factor to negatively regulate miR-211-5p and miR-377-3p in CC cells.

### Circ_0000118 Controls the Malignant Phenotypes of CC Cells Through Modulating miR-211-5p/miR-377-3p

**Fig. 4:**
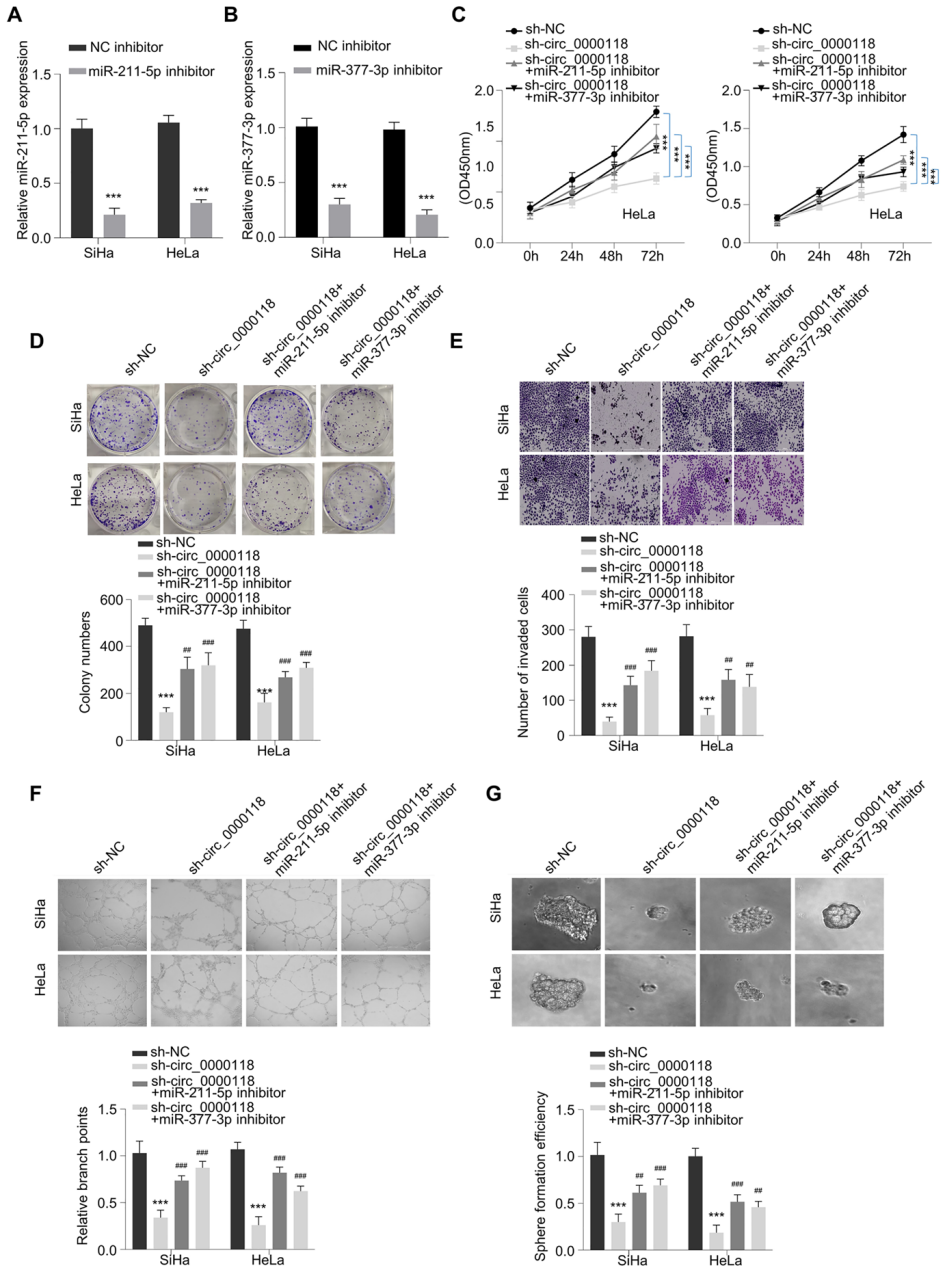
Circ_0000118 controls the malignancy of CC cells by sponging miR-211-5p/miR-377-3p. **A** miR-211-5p levels in CC cells were detected upon the transfection of miRNA inhibitor or inhibitor NC (negative control of the inhibitor). **B** miR-377-3p levels in CC cells after the transfection of miRNA inhibitor or inhibitor NC (negative control of the inhibitor). **C** CCK-8 proliferation assay was conducted at 0 h, 24 h, 48 h, and 72 h in CC cells with different treatments. **D** Colony generation experiment in CC cells after different treatments. **E** Cell invasiveness assessment in CC cells after different treatments. **F** Angiogenesis assay in CC cells after different treatments. **G** 3D tumor sphere assay in CC cells after indicated treatments. **P < 0.01, ***P < 0.001

Next, we sought to examine the involvement of miR-211-5p/miR-377-3p in the regulatory effect of circ_0000118 in CC cells. First, the transfection with corresponding miRNA inhibitor downregulated the miRNAs in cervical cancer cells (Fig. 4A, B). As revealed by CCK-8 assay, both miRNA inhibitors were able to rescue the detrimental effects of circ_0000118 knockdown on cell growth (Fig. 4C). Similarly, the application of miR-211-5p or miR-377-3p inhibitor also mitigated the effects of circ_0000118 knockdown on colony formation (Fig. 4D), cell invasiveness (Fig. 4E), tube formation ability (Fig. 4F), and cell growth in the 3D sphere assay (Fig. 4G). Therefore, these data indicate that circ_0000118 modulates the malignancy of CC cells by regulating miR-211-5p and miR-377-3p activities.

### AKT2 is Downregulated by miR-211-5p and miR-377-3p in CC Cells

**Fig. 5:**
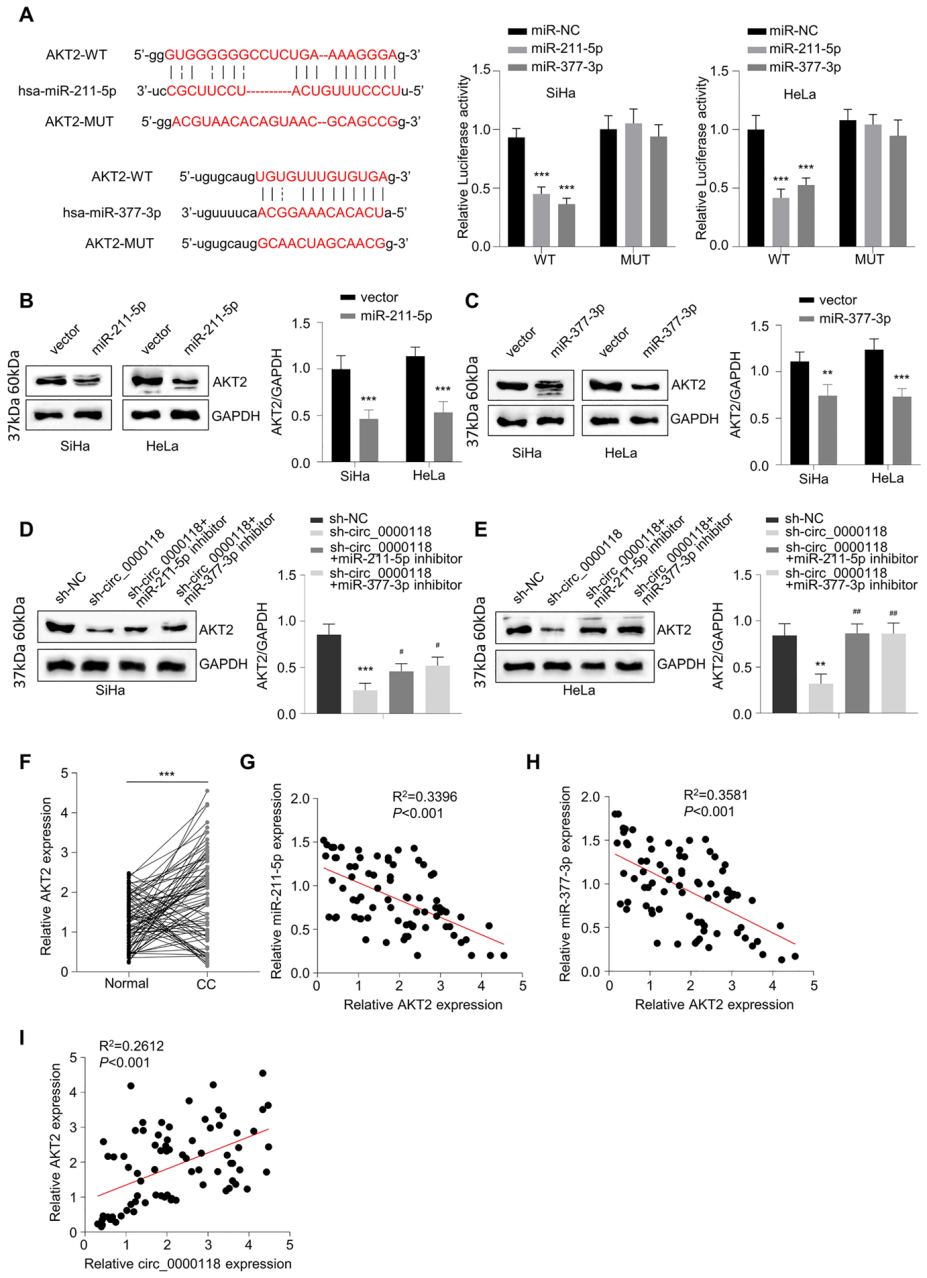
AKT2 is downregulated via miR-211-5p and miR-377-3p in CC. **A** According to Starbase database analysis, there are miR-211-5p and miR-377-3p interaction sites in AKT2 mRNA UTR, and dual-luciferase reporter assay validated their functional interactions in CC cells. **B**, **C** AKT2 protein measurement in CC cells after the transfection of miR-211-5p or miR-377-3p mimic. **D**, **E** AKT2 protein levels in CC cells with indicated treatment groups. **F** AKT2 mRNA levels in 78 CC patient samples and healthy controls. **G**, **H** Correlative analysis of AKT2 mRNA and miR-211-5p or miR-377-3p level in 78 CC patient samples. **I** Correlative analysis of circ_0000118 and AKT2 mRNA level in 78 CC patient samples. **P < 0.01, ***P < 0.001

According to Starbase online analysis, there were several possible mRNA targets of miR-211-5p and miR-377-3p (Fig. S2A). RNA pull-down experiment showed that miR-211-5p and miR-377-3p could heavily enrich AKT2 mRNA in HeLa cells (Fig. S2B). The miR-211-5p and miR-377-3p’s interaction sites within the 3′ untranslated region of AKT2 mRNA were shown, and dual-luciferase activity data revealed that miR-211-5p or miR-377-3p overexpression could inhibit the WT reporter containing wild-type sequence of AKT2 3′-UTR in CC cells (Fig. 5A). Upregulating miR-211-5p or miR-377-3p level could reduce AKT2 protein level (Fig. 5B, C). Knockdown of circ_0000118 also reduced AKT2 protein level, which was attenuated after the co-transfection of miR-211-5p or miR-377-3p inhibitor (Fig. 5D, E). In the meanwhile, AKT2 protein level was markedly upregulated in CC patient samples (Fig. 5F), and AKT2 mRNA level displayed negative correlations with miR-211-5p and miR-377-3p level (Fig. 5G, H), but showed a positive correlation with circ_0000118 level in CC samples (Fig. 5I). These data indicate that AKT2 mRNA is negatively targeted by miR-211-5p and miR-377-3p in CC cells.

### Overexpression of AKT2 Partially Reverses the Detrimental Effects of Knocking Down Circ_0000118

**Fig. 6:**
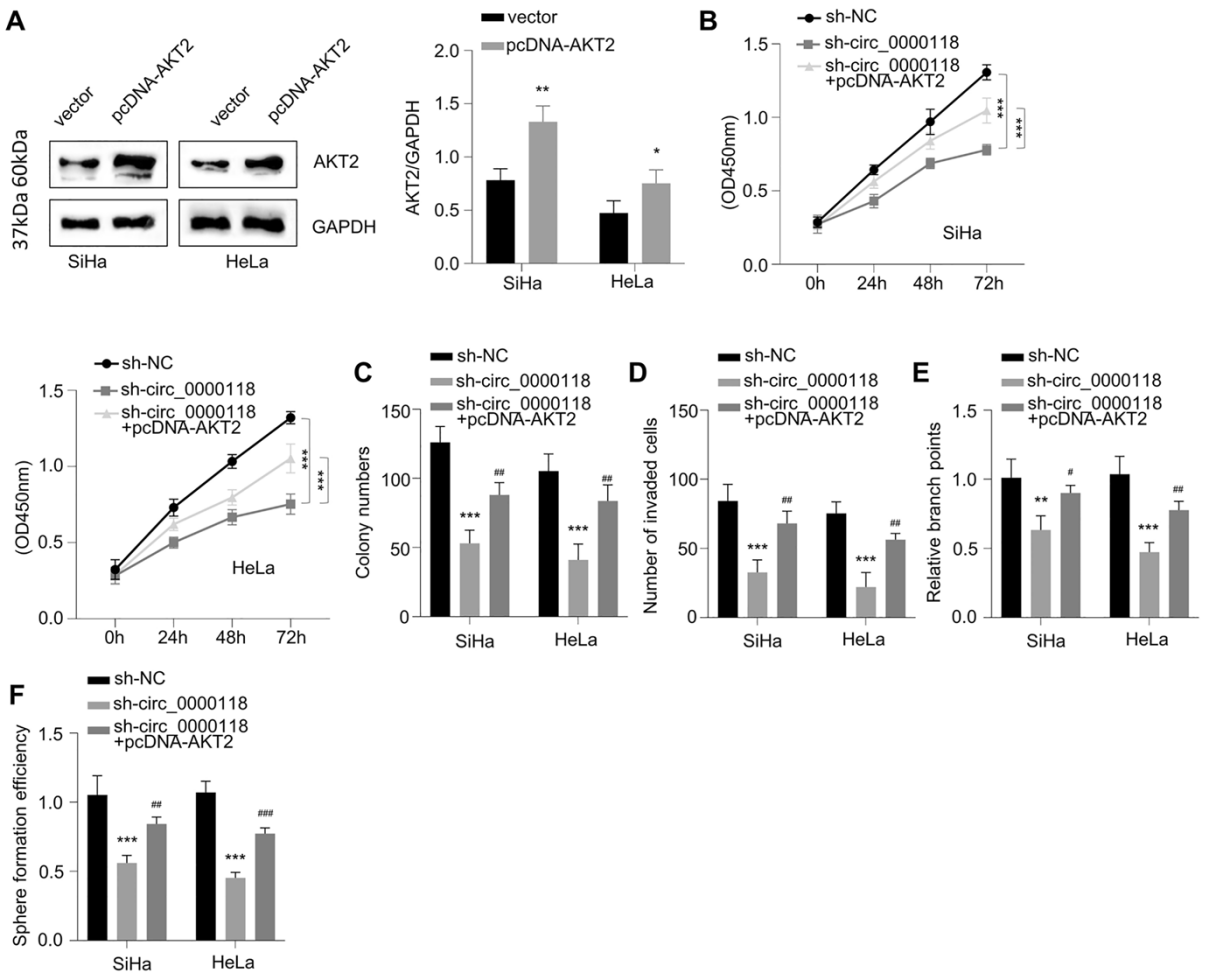
Upregulating AKT2 partially abolishes circ_0000118’s inhibition on CC cells. **A** AKT2 protein level measurement in CC cells transfected with empty vector or AKT2 expression vector (pcDNA-AKT2). **B** CCK-8 cell growth assay was conducted at 0 h, 24 h, 48 h, and 72 h in CC cells of different experimental groups. **C** Colony formation experiment in CC cells of different experimental groups. **D** Cell invasiveness assay in CC cells of different experimental groups. **E** Angiogenesis assay in CC cells of different experimental groups. **F** 3D tumor sphere assay in CC cells of different experimental groups. **P < 0.01, ***P < 0.001

To confirm that AKT2 is involved in the regulatory role of circ_0000118, we transfected CC cells with empty plasmid or AKT2 expression vector (pcDNA-AKT2). Cells transfected with pcDNA-AKT2 showed a significant elevation of AKT2 protein level (Fig. 6A). Knockdown of circ_0000118 reduced cell proliferation capacity, which was partially restored by AKT2 overexpression (Fig. 6B). In addition, the inhibitory effects of circ_0000118 knockdown on the colony generation capacity (Fig. 6C), cell invasiveness (Fig. 6D), tube formation ability (Fig. 6E), and tumor sphere formation (Fig. 6F) were also partially rescued by AKT 2 overexpression. These data suggest that AKT2 mediates the downstream effects of circ_0000118 in CC cells.

### Knocking Down Circ_0000118 Suppresses the Tumorigenesis of CC Cells

**Fig. 7:**
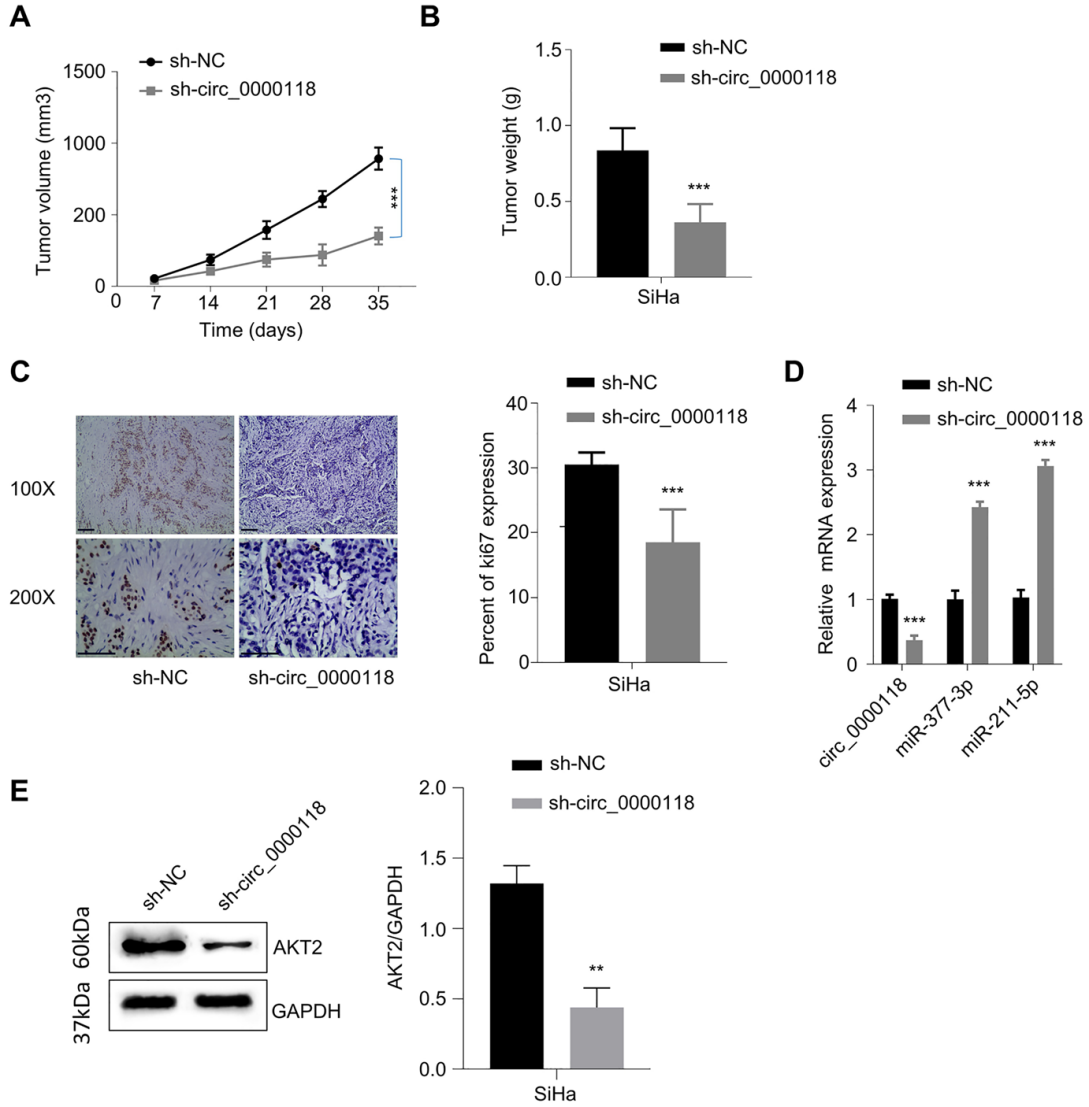
Knocking down circ_0000118 inhibits tumor formation of CC cells in vivo. **A** and **B** The volume and weight of xenograft tumors were measured in sh-NC and sh-circ_0000118 groups (n = 6 in each group). **C** Ki-67 IHC analysis in the tissue sections of sh-NC and sh-circ_0000118 tumors. **D** qRT-PCR detection of circ_0000118, miR-211-5p, and miR-377-3p in subcutaneous xenograft tissues of each experimental group. **E** AKT2 protein levels in subcutaneous xenograft tissues of each. *P < 0.05, **P < 0.01, ***P < 0.001

We further evaluated the effect of circ_0000118 silencing in the tumorigenesis. SiHa cells expressing sh-NC (negative control shRNA) or sh-circ_0000118 (sh-RNA targeting circ_0000118) were inoculated into nude mice as xenograft model. The results showed that silencing circ_0000118 delayed tumor formation, as revealed by the lowered tumor volume and weight after circ_0000118 knockdown (Fig. 7A, B). IHC staining showed that circ_0000118 knockdown reduced Ki-67 expression level in the xenograft tumor tissues (Ki-67 is a cellular marker of cell proliferation) (Fig. 7C). Besides, according to qRT-PCR assay, sh-circ_0000118 significantly upregulated miR-211-5p and miR-377-3p in the xenograft tumor tissues (Fig. 7D), but decreased the expression level of AKT2 (Fig. 7E). These results further corroborated that circ_0000118 functions as a tumor-promoting circRNA and augments the malignancy of CC cells.

## Discussion

Emerging roles of circRNAs in cancer development have attracted intensified research interest. It has been reported that hsa_circRNA_101996 can induce TPX2 (TPX2 Microtubule Nucleation Factor) expression through suppressing miR-8075, which mediates the cell division and migratory phenotype in CC (Song et al. 2019). Besides, circEIF4G2 was reported to enhance the malignant phenotype of CC cells by modulating mirR-218/HOXA1 (Homeobox A2) axis (Mao et al. 2019). In addition, circSMARCA5 and hsa_circ_0008285 are also implicated in the malignancy regulation of CC cells (Tian and Liang 2018). Here, we showed an upregulation of circ_0000118 in CC patient samples and cells. Furthermore, silencing circ_0000118 largely impaired the malignant characteristics of CC cells and repressed the tumorigenesis. According to the above results, we propose that circ_0000118 may serve as an oncogenic circRNA during the progression of cervical cancers.

MiRNAs are the first type of non-coding RNAs implicated in tumor pathology (Lee and Dutta 2009). CircRNAs have been suggested to exert roles of ceRNAs (competitive endogenous RNAs) to sponge miRNAs and regulate their downstream targets (Qi et al. 2015). The interactions between miRNAs with circRNAs can regulate the malignancy and aggressiveness in cancer cells (Lee and Dutta 2009; Qi et al. 2015). The present work demonstrated that circ_0000118 could target both miR-211-5p and miR-377-3p. As reported by Bai and colleagues, miR-211-5p showed downregulation in CC, which can regulate the growth and invasiveness in CC cells by targeting SOX4 (SRY-Box Transcription Factor 4) (Bai and Li 2020). miR-377-3p was also reported to be downregulated in CC samples, and it can regulate the malignancy in CC cells by repressing the level of SGK3 (Serum/Glucocorticoid Regulated Kinase Family Member 3) (Zhang et al. 2020). Consistent with the above studies, we showed the downregulation of both miR-211-5p and miR-377-3p in CC cells. The interaction of circ_0000118 and these two miRNAs regulated the malignant characteristics in CC cells. Based on these data, we conclude that circ_0000118 exerts the carcinogenic effect in CC by repressing the activity of miR-211-5p and miR-377-3p.

According to our findings, miR-211-5p and miR-377-3p mimics remarkably downregulated AKT2 protein level. miR-211-5p or miR-377-3p overexpression reduced the WT reporter activity containing AKT2 mRNA 3′UTR, suggesting that AKT2 mRNA can be physically targeted by miR-211-5p and miR-377-3p. In addition, we further showed that AKT2 overexpression rescued the detrimental effects of circ_0000118 silencing in CC cells. These findings support that AKT2 promotes the progression of CC by acting as a downstream mediator of circ_0000118. Consistently, a previous study showed that AKT2 level was increased in CC tumor samples. Downregulating AKT2 suppressed cell proliferation and survival in CC cells (Zhao et al. 2020). We further unveiled that AKT2 was directly targeted by both miR-211-5p and miR-377-3p, while circ_0000118 could positively regulate AKT2. These data suggest that circ_0000118 serves as an upstream regulator to maintain the expression of AKT2 through sponging miR-211-5p and miR-377-3p. However, it is worth mentioning that AKT2 mRNA could be modulated by multiple miRNAs (Wu et al. 2019). The crosstalk of different miRNAs in the regulation of AKT2 mRNA stability and translation needs to be further clarified.

There are several questions needing to be further clarified based on our data. First, how circ_0000118 becomes dysregulated in CC is unclear. The answer to this question is the key to manipulating circ_0000118 for anti-cancer purpose. In addition, other downstream targets of circ_0000118 remain to be identified and characterized. Perhaps a genome-wide transcriptome analysis could shed light on the competitive endogenous RNA (ceRNA) network governing by circ_0000118. These information would provide a holistic picture of the signaling and cellular processes regulated by circ_0000118.

Taken together, the present work revealed an oncogenic effect of circ_0000118 in the tumorigenesis and malignancy of CC. Circ_0000118 silencing largely attenuated the aggressive characteristics of CC cells and repressed the tumorigenesis in xenograft mouse model. We further demonstrated that miR-211-5p and miR-377-3p as downstream targets of circ_0000118. Circ_0000118 competitively sponges miR-211-5p and miR-377-3p to release the inhibition on AKT2 and maintain its protein level for the support of malignant proliferation in CC cells. These data laid the theoretical foundations for therapeutic management in CC by targeting circ_0000118/miR-211-5p and miR-377-3p/AKT2 axis.

## Supplementary Information

Below is the link to the electronic supplementary material.

Supplementary file1 (JPG 1071 KB). Fig. S1. (A). Starbase prediction of the potential miRNA targets of circ_0000118. (B). RNA pull down analyses of the predicted miRNA targets using circ_0000118 biotin-labeled probe in HeLa cells. Data were normalized to the control probe.

Supplementary file2 (JPG 1321 KB). Fig S2. (A) Possible mRNA targets of miR-211-5p and miR-377-3p predicted by Starbase. (B) RNA pull-down analysis of mRNA targets using miR-211-5p and miR-377-3p biotin probes or control probe in HeLa cells. ***P<0.001.

## Data Availability

All the data generated and/or analyzed during this study are available upon email request to the corresponding author.
