# Supplementary figures and images for: CircRNA Circ_0000118 Regulates Malignancy of Cervical Cancer Cells by Regulating miR-211-5p/miR-377-3p/AKT2 Axis
Source: Biochem Genet. 2023 Jan 31;61(4):1625–44. doi: 10.1007/s10528-023-10332-w (PMC10371915; doi:10.1007/s10528-023-10332-w)

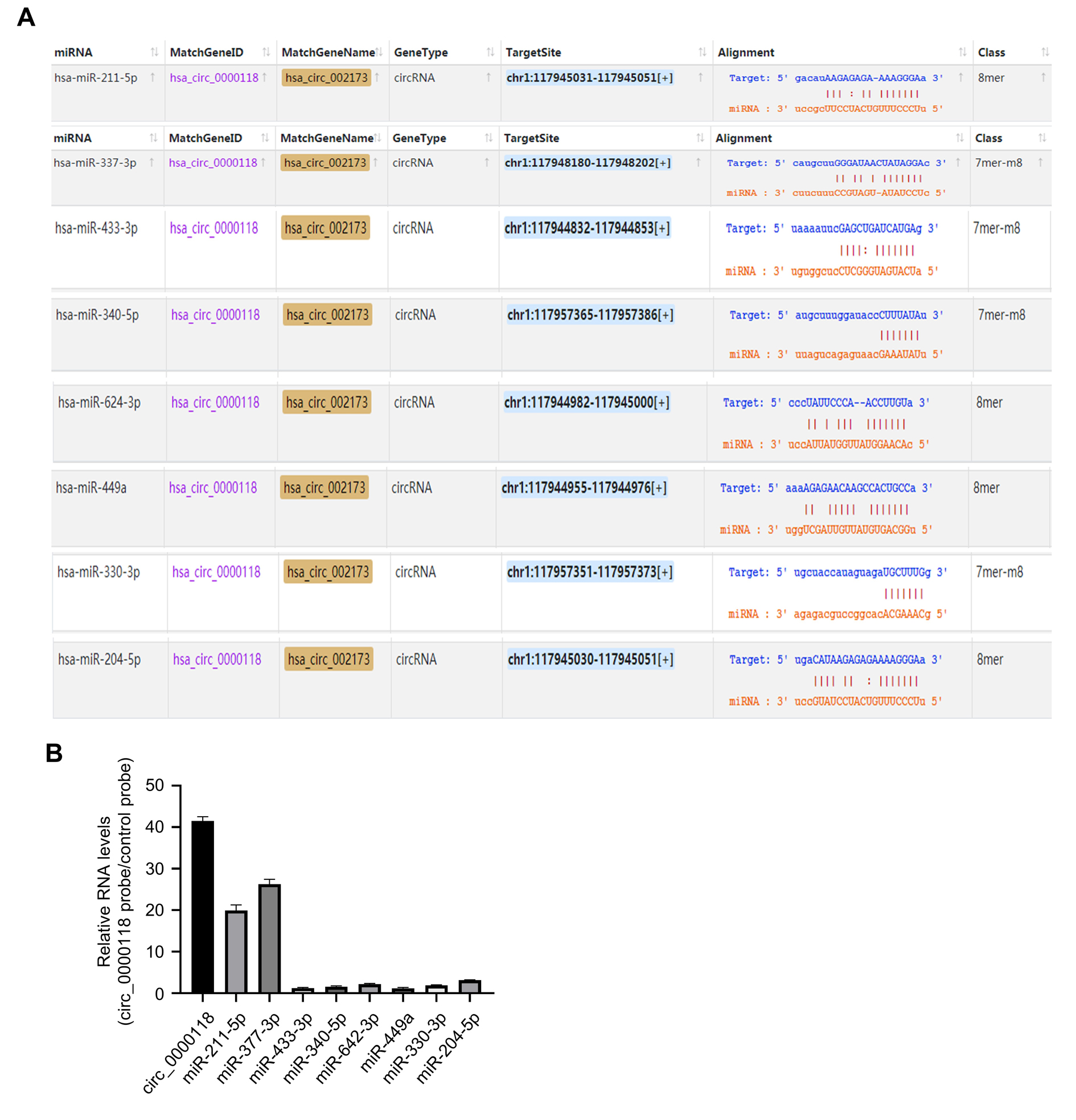

Supplement: Supplementary file 1 — Supplementary file1 (JPG 1071 KB). Fig. S1. (A). Starbase prediction of the potential miRNA targets of circ_0000118. (B). RNA pull down analyses of the predicted miRNA targets using circ_0000118 biotin-labeled probe in HeLa cells. Data were normalized to the control probe. [file 10528_2023_10332_MOESM1_ESM.jpg]

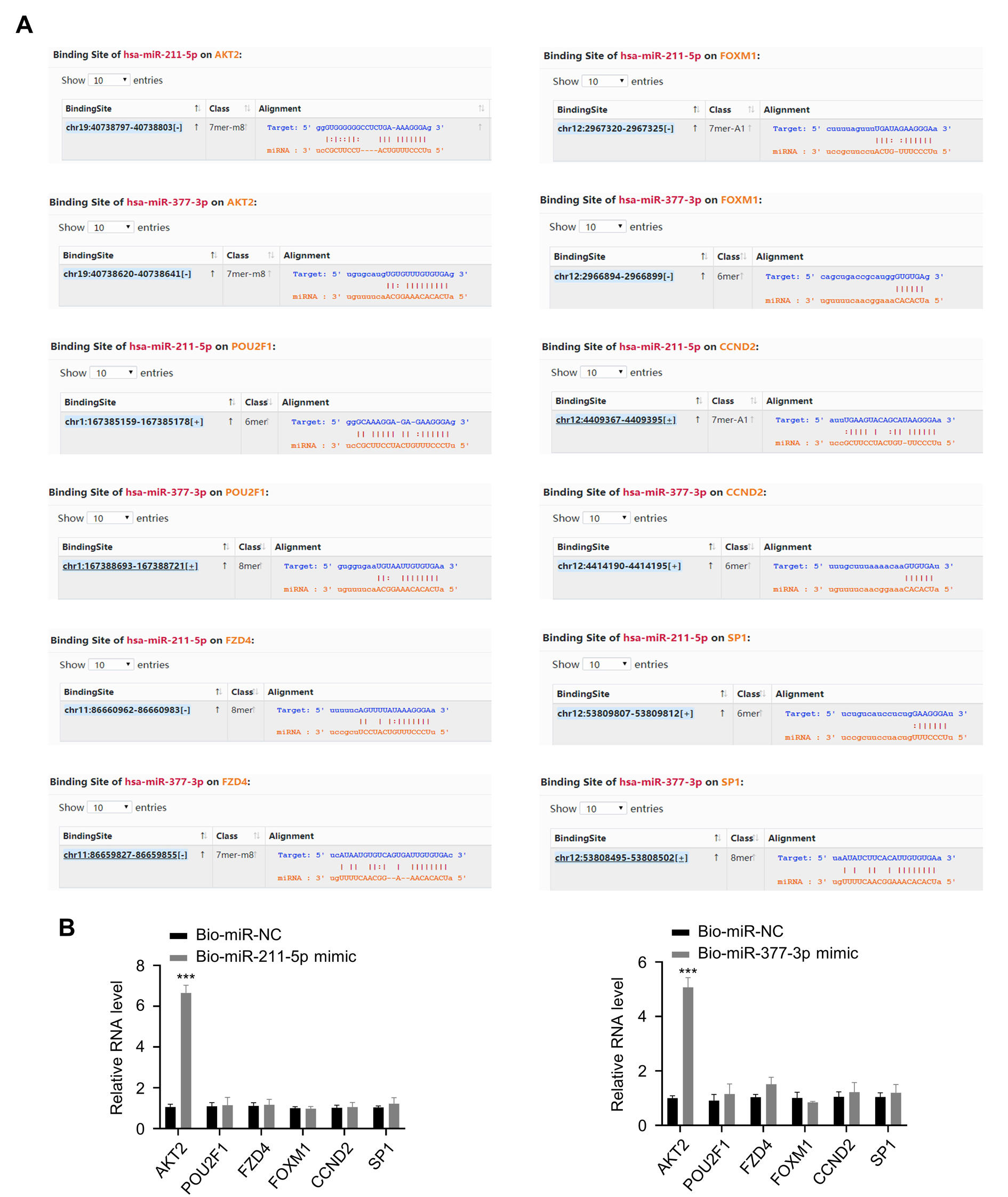

Supplement: Supplementary file 2 — Supplementary file2 (JPG 1321 KB). Fig S2. (A) Possible mRNA targets of miR-211-5p and miR-377-3p predicted by Starbase. (B) RNA pull-down analysis of mRNA targets using miR-211-5p and miR-377-3p biotin probes or control probe in HeLa cells. ***P<0.001. [file 10528_2023_10332_MOESM2_ESM.jpg]
